# Do US quitsites present information related to providing services for LGBTQ individuals? An audit study

**DOI:** 10.18332/tpc/191457

**Published:** 2024-08-29

**Authors:** Jeffrey Wilmer Ramos-Santiago, Scott McIntosh, Rafael H. Orfin, Daimarelys Lara, Skylar Joseph, Manpreet Kaur, Alixida Ramos-Pibernus, Ana Paula Cupertino, Deborah J. Ossip, Francisco Cartujano-Barrera

**Affiliations:** 1 Department of Public Health Sciences, University of Rochester, New York, United States; 2 School of Behavioral and Brain Sciences, Ponce Health Sciences University, Ponce, Puerto Rico; 3 Department of Surgery, University of Rochester, New York, United States

**Keywords:** LGBTQ, services, audit, quitsites, quitlines

## Abstract

**INTRODUCTION:**

Most US quitlines have quitsites and websites designated to promote their services. Quitsites have the potential to encourage LGBTQ individuals to utilize quitline services by explicitly mentioning the provision of LGBTQ-competent services. The present study audited quitsites to determine the presence of information regarding services for LGBTQ individuals.

**METHODS:**

Using a checklist consisting of nine criteria, a cross-sectional audit of the US quitsites was conducted between 16 October and 8 November 2023. The audit was divided into two phases: 1) auditors coded all quitsites separately, and 2) auditors met with the first author to compare their coding and reach a consensus. The inter-rater agreement was calculated. Frequencies and percentages were calculated for each criterion.

**RESULTS:**

Auditors evaluated a total of 46 quitsites. Inter-rater agreement was 96.85%. Seven quitsites (15.2%) met 0 of the nine criteria, and 36.9% of the quitsites (17/46) met more than six criteria. Only one quitsite met 8 of 9. No individual website met all nine criteria. While 84.8% of quitsites had at least a singular mention of the LGBTQ community somewhere on their website, only 4.3% of the quitsites mentioned the LGBTQ community on their landing page.

**CONCLUSIONS:**

Most quitsites mentioned the LGBTQ community somewhere on their website (84.8%). However, only 4.3% of the quitsites mentioned the LGBTQ community on their landing page. Results suggest that quitsites explicitly mention the provision of services for LGBTQ individuals on their landing page, which has the potential to engage LGBTQ individuals into quitline services and reduce tobacco-related disparities.

## INTRODUCTION

Approximately 20 million adults (8% of the adult population) in the US, self-identify as lesbian, gay, bisexual, transgender, or queer (LGBTQ)^1^. In the US, LGBTQ individuals have higher rates of tobacco use compared to heterosexual, cisgender individuals^1-3^. For example, data from the 2021 CDC Morbidity and Mortality Weekly Report (MMWR) show that 15.3% of LGB adults smoke cigarettes, compared to 11.4% of heterosexual adults^3^. Similarly, data from the 2016–2018 Population Assessment of Tobacco and Health (PATH) study, reported that transgender individuals were 2 to 3 times more likely to use tobacco compared to cisgender individuals^4^. Importantly, LGBTQ individuals are as likely to want to quit tobacco as heterosexual, cisgender individuals. However, the utilization of evidence-based tobacco cessation treatments is very low among LGBTQ individuals^5^.

Quitlines are an effective treatment option that supports tobacco cessation in the US^6-7^. Quitlines are state-funded, free-of-charge programs that provide confidential tobacco cessation counseling over the phone for all individuals living in the US^7-9^. Some quitlines offer additional support, such as smoking cessation medications (e.g. nicotine patches) and text messaging services. A recent study reported that in California, 7% of the individuals who utilized the quitline were part of the LGBTQ community^10^. In comparison, 9.1% of Californians consider themselves part of the LGBTQ community^10^. Notably, LGBTQ individuals who utilized the quitline had similar cessation rates compared to heterosexual, cisgender individuals. Reducing tobacco-related disparities among LGBTQ individuals depends greatly on engaging them in a tobacco cessation treatment (e.g. quitline services)^11^.

Most US quitlines have quitsites and websites designated to promote their services. Quitsites have the potential to encourage LGBTQ individuals to utilize quitline services by explicitly mentioning the provision of LGBTQ-competent services. Such information is relevant because LGBTQ individuals often report not seeking tobacco cessation services due to fear of mistreatment^12^. The present study audited US quitsites for the presence of information regarding services for LGBTQ individuals.

## METHODS

This was a cross-sectional audit study of the US quitsites. The first and last authors developed an audit checklist that consisted of nine criteria assessing whether the quitsites presented information related to providing services for LGBTQ individuals. These criteria were developed according to recommendations for website design and user engagement^13^. The first author pilot-tested the audit checklist in September 2023. The results of the pilot test were reviewed with the last author, and final refinements to the audit checklist were made. Table 1 outlines the final audit checklist and each criterion’s definition.

**Table 1:** Nine checklist criteria and definitions developed for LGBTQ audit of US quitsites, 2023

*Criteria*	*Definitions*
Uses LGBTQ symbology on quitsite	The quitline website used symbols (e.g. pink triangles, interlocking female and/or male gender symbols) or imagery (e.g. rainbows, pride flags) associated with LGBTQ individuals.
Mentions LGBTQ on the quitsite landing page	The quitline website made any explicit use of the ‘LGBTQ’ term and/or terms that refer to non-cisnormative and non-heteronormative sexual and/or gender identities on the landing page (i.e. a web page accessed by clicking on a quitline logo or advertisement, and designed to persuade users to take a specific action, such as browse around or sign up). This does not include drop-down navigation bars.
Mentions LGBTQ on the quitsite general webpage	The quitline website made any explicit use of the ‘LGBTQ’ term and/or terms that refer to non-cisnormative and non-heteronormative sexual and/or gender identities on any part of the page. This may include drop-down navigation bars.
Has a separate LGBTQ page	The quitline website contained a separate page to highlight topics, resources, and issues associated with tobacco cessation among LGBTQ individuals.
Uses LGBTQ fact sheet(s)	The quitline website provided a single-page document and/or handout with essential information about tobacco cessation for LGBTQ individuals. Essential information may include use rates, graphics/tables, and health risks and outcomes for LGBTQ individuals who use tobacco. Fact sheets describing program services exclusively are not included.
Provides links to LGBTQ resources	The quitline web page redirects the user to any resource specific to LGBTQ individuals, whether internal or external, to the quitline program.
Facilitates LGBTQ news/articles	The quitline website published and/or provided links to educational articles and/or informative news on topics linked to LGBTQ individuals and tobacco.
Mentions LGBTQ tobacco use rates	The quitline website presents data related to tobacco use among LGBTQ individuals.
Mentions LGBTQ vulnerability	The quitline website mentions any data that alludes to the documented vulnerability that LGBTQ individuals have to tobacco use.

The audit was conducted between 6 October and 8 November 2023. The audited quitsites were the official quitsites of the 50 states and Washington, D.C., listed on the North American Quitline Consortium (NAQC) website (https://map.naquitline.org/reports/web/). States that did not have a quitsite specific to their quitline were excluded from the analysis. Moreover, quitsites with multiple websites were counted as one quitsite. The third and fourth authors, who will be referred to as auditors for the remainder of the article, conducted the audit. The first author trained the auditors on using the audit checklist before beginning the data collection. The audit was divided into two phases. In the first phase, auditors coded all quitsites independently. Then, both auditors sent their checklists to the first author to compare their coding and identify points of disagreement. In the second phase, auditors met with the first author to discuss points of disagreement and reach a consensus. Lastly, each quitsite was assigned a random number to blind their identity.

The inter-rater agreement (number of agreements over the number of opportunities for agreement)^14^ was calculated at the end of the first phase. Frequencies and percentages were calculated for categorical variables.

## RESULTS

Five states did not have a quitsite specific to their quitline: two relied on their state government public health website to include the quitline contact information and three directed users to the US national quitsite. Moreover, seventeen quitsites had a second website that could be accessed from the first. Hence, only 46 quitsites were evaluated. The inter-rater agreement was 96.85%.

Table 2 outlines the results of the audit. Seven quitsites (15.2%, 7/46) met 0 of the 9 criteria. About a third of the quitsites (36.9%, 17/46) met more than six criteria. Only one quitsite – the Colorado quitsite – met 8 of 9. No individual website met all nine criteria.

**Table 2:** Results of the LGBTQ audit of the US quitsites, 2023 (N=46)

STATE*	*LGBTQ+ symbology on quitline profile*	*Mentions LGBTQ+ on quitsite landing page*	*Mentions LGBTQ+ on quitsite general website*	*Separate LGBTQ+ page*	*LGBTQ+ fact sheet*	*Links to LGBTQ+ resources*	*LGBTQ+ news/articles*	*Mention LGBTQ+ tobacco usage rates*	*Mentions LGBTQ+ vulnerability*	*n (%)*
**1**	X		X	X		X	X	X	X	7 (77.7)
**2**	X		X	X			X	X	X	6 (66.6)
**3**			X				X	X	X	4 (44.4)
**4**	X		X	X			X	X	X	6 (66.6)
**5**	X		X	X			X	X	X	6 (66.6)
**6**	X		X	X				X	X	5 (55.5)
**7**	X		X	X			X	X	X	6 (66.6)
**8**			X			X	X		X	4 (44.4)
**9**			X			X				2 (22.2)
**10**	X		X		X		X	X	X	6 (66.6
**11**			X			X	X		X	4 (44.4)
**12**			X							1 (11.1)
**13**		X	X					X		3 (33.3)
**14**										0 (0)
**15**		X	X	X		X		X	X	6 (66.6)
**16**	X		X							2 (22.2)
**17**										0 (0)
**18**										0 (0)
**19**			X						X	2 (22.2)
**20**	X		X	X			X	X	X	6 (66.6)
**21**										0 (0)
**22**	X		X	X			X	X	X	6 (66.6)
**23**	X		X			X		X	X	5 (55.5)
**24**			X		X			X	X	4 (44.4)
**25**	X		X			X				3 (33.3)
**26**	X		X			X		X	X	5 (55.5)
**27**	X		X	X			X	X	X	6 (66.6)
**28**			X						X	2 (22.2)
**29**	X		X	X			X	X	X	6 (66.6)
**30**										0 (0)
**31**	X		X	X		X	X	X	X	7 (77.7)
**32**			X	X		X		X	X	5 (55.5)
**33**	X		X	X			X	X	X	6 (66.6)
**34**										0 (0)
**35**	X		X	X		X	X	X	X	7 (77.7)
**36**										0 (0)
**37**	X		X	X			X	X	X	6 (66.6)
**38**	X		X	X			X	X	X	6 (66.6)
**39**			X					X		2 (22.2)
**40**										0 (0)
**41**	X		X	X			X	X	X	6 (66.6)
**42**	X		X	X			X	X	X	6 (66.6)
**43**	X		X	X			X	X	X	6 (66.6
**44**			X							1 (11.1)
**45**	X		X	X			X	X	X	6 (66.6)
**Colorado**	X		X	X	X	X	X	X	X	8 (88.8)
**n (%)**	25 (54.3)	2 (4.3)	39 (84.8)	22 (47.8)	3 (6.5)	12 (26.0)	23 (50)	29 (63.0)	32 (69.5)	

* Simple numerical order.

Just over half of the quitsites (54.3%, 25/46) used symbols or images associated with LGBTQ individuals. Most quitsites (84.8%, 39/46) mentioned the LGBTQ community somewhere on their website. However, only 4.3% (2/46) of the quitsites mentioned the LGBTQ community on their landing page. Just under half (47.8%, 22/46) of quitsites linked users to a separate page about the LGBTQ community. A few (6.5%, 3/46) had fact sheets with specific information about the LGBTQ community. About one-quarter of the quitsites (26.0%, 12/46) provided links to resources specific to LGBTQ individuals. Half of the quitsites (50%, 23/46) had news articles about tobacco use among LGBTQ individuals. Lastly, more than half of the quitsites included statistics on tobacco use among LGBTQ individuals (63.0%) and alluded to the unique vulnerabilities that LGBTQ individuals have to tobacco (69.5%).

## DISCUSSION

This study assessed whether US quitsites present information relevant to providing services for LGBTQ individuals. Our findings indicated that most quitsites mentioned the LGBTQ community somewhere on their website (84.8%), with a range of LGBTQ informational resources offered across quitsites. However, only 4.3% of the quitsites mentioned the LGBTQ community on their landing page. This result is important because of the following reasons: 1) previous studies have reported that messages from tobacco control programs serving LGBTQ individuals must be explicitly LGBTQ-friendly or risk being interpreted as exclusionary and not applicable to the LGBTQ community^11^; and 2) websites often have high bounce rates (i.e. the act of visiting a webpage without exploring other pages on a given site)^15,16^. It is recommended that quitsites explicitly mention on their landing page the provision of services for LGBTQ individuals, which has the potential to engage LGBTQ individuals into quitline services and reduce tobacco-related disparities.

Some quitsites presented information related to providing services for LGBTQ individuals in clickable website elements under ambiguous titles and/or images. For example, multiple websites had a clickable website element named ‘Tobacco and You’, which then included information about LGBTQ individuals. Furthermore, one of the websites presented printable material (i.e. a ‘palm card’) named ‘LGBTQ – palm card’. The printable material included a picture of different individuals in front of what appeared to be a pride flag. However, the individuals mostly covered the flag and only showed three colors (i.e. red, yellow, and green). Moreover, the printable material did not explicitly mention ‘LGBTQ’. These results highlight the need for quitsites to mention their services for LGBTQ individuals in an upfront and explicit manner.

Although the study audited publicly available websites, the authors decided to mask state identities. The only exception was the identity of the Colorado quitsite, which performed well in the audit and set a clear example of the information that quitsites should present concerning providing services for LGBTQ individuals. The rationale for masking state identities was to audit all US quitsites without being critical of individual state efforts. To make sure that quitsites present information related to providing services for LGBTQ individuals, the authors invite all state quitlines to contact our research team if they want to know how their quitsite performed in the audit.

We acknowledge that LGBTQ individuals are not the only group who experiences tobacco-related disparities^17^. This study purposely focused on LGBTQ individuals and, as such, is solely drawing conclusions and recommendations for this group. Future research is needed to audit US quitsites for the presence of information regarding competent services for other groups who experience tobaccorelated disparities (e.g. Black, Native American, and Latino individuals).

### Strengths and limitations

This study has some strengths that are worth mentioning. First, two auditors conducted the audit systematically and independently using a detailed audit checklist. Second, the audit was conducted in a short window of time, which reduced the possibility of website changes during the study due to periodic updates. Lastly, our research team includes members of the LGBTQ community whose experiences helped to inform the development of the audit checklist, guided the evaluation process that resulted in an appropriate inter-rater agreement, and allowed for a consensus to be reached among auditors.

This study has some limitations. First, this was a cross-sectional audit. Like any other website, quitsites are updated periodically; hence, the results of this audit are limited to the study period. Second, this audit focused on whether the quitsites met the checklist criteria, not the quality of implementation of each criterion. Future studies should focus on understanding the perceptions of LGBTQ individuals on the quality of quitsites and the information these websites provide them. Lastly, the audit was conducted exclusively in English. Whether and the extent to which the quitsites present information regarding services for LGBTQ individuals in other languages (e.g. Spanish) was not determined.

## CONCLUSIONS

Most quitsites mentioned the LGBTQ community somewhere on their website (84.8%). However, only 4.3% of the quitsites mentioned the LGBTQ community on their landing page. It is recommended that quitsites explicitly mention the provision of services for LGBTQ individuals on their landing page, which has the potential to engage LGBTQ individuals into quitline services and reduce tobacco-related disparities.

## Data Availability

The data generated in this study are available upon request from the corresponding author.
